# Embryonic stem cells reduce liver fibrosis in CCl_4_-treated mice

**DOI:** 10.1111/j.1365-2613.2008.00607.x

**Published:** 2008-12

**Authors:** Kei Moriya, Masahide Yoshikawa, Yukiteru Ouji, Ko Saito, Mariko Nishiofuku, Ryosuke Matsuda, Shigeaki Ishizaka, Hiroshi Fukui

**Affiliations:** * Third Department of Internal Medicine, Nara Medical University Kashihara, Nara, Japan; † Department of Parasitology, Nara Medical University, Kashihara Nara, Japan

**Keywords:** carbon tetrachloride, DlK-1, embryonic stem cells, hepatoblast, intrasplenic transplantation, liver fibrosis, metalloproteinase

## Abstract

We transplanted undifferentiated embryonic stem (ES) cells into the spleens of carbon tetrachloride (CCl_4_)-treated mice to determine their effects on liver fibrosis. Carbon tetrachloride at 0.5 ml/kg of body weight was injected intraperitoneally into C57BL/6 mice twice weekly for up to 20 weeks. Four weeks after the first injection, the mice were divided into two groups and those in group 1 received 1 × 10^5^ ES cells genetically labelled with enhanced green fluorescent protein (GFP) in the spleens, while group 2 mice received 0.1 ml of phosphate-buffered saline. In group 1, GFP-immunopositive cells were retained and found in areas of fibrosis in the liver, and reduced liver fibrosis was observed as compared with group 2. Secondary transplantation of ES cells at 12 weeks after the initial transplantation enhanced the reduction in liver fibrosis. No teratoma formation or uncontrolled growth of ES cells in organs, including the liver and spleen, was observed in any of the mice. In the livers of group 1 mice, metalloproteinase 9-immunopositive cells derived from ES cells as well as those from the recipient were observed. These cells were also found to be immunopositive for the hepatoblast marker Delta-like (DlK-1), a member of the DlK-1 family of transmembrane proteins. These results suggest that ES-based cell therapy is potentially useful for liver fibrosis treatment and that reduction in CCl_4_-induced liver fibrosis by transplantation of ES cells may be related closely to the emergence of metalloproteinase-producing hepatoblast-like cells.

Liver cirrhosis is one of the most representative forms of liver fibrosis and represents a serious health problem. Recently, transplantation of bone marrow-derived cells including mesenchymal stem cells was reported to reduce carbon tetrachloride (CCl_4_)-induced liver fibrosis (Fang *et al.* 2004; Sakaida *et al.* 2004; Wu *et al.* 2005; Zhao *et al.* 2005; Oyagi *et al.* 2006), while foetal liver epithelial progenitor cells have also been shown to ameliorate diethylnitrosamine-induced liver fibrosis (Zheng *et al.* 2006). Thus, transplantation of cells carrying the characteristics of stem cells or progenitor cells seems to improve liver fibrosis.

Embryonic stem (ES) cells are self-renewing and pluripotent cells derived from the inner cell masses of preimplantation blastocysts (Evans & Kaufman 1981; Martin 1981), and are considered to be an optimal source for cell-replacement therapy, because of their limitless growth and pluripotent differentiation capacity. We previously reported the differentiation of mouse ES cells into dopaminergic neurons (Nishimura *et al.* 2003), rhodopsin-immunopositive photoreceptor-like cells (Sugie *et al.* 2005), insulin-secreting cells (Shiroi *et al.* 2002), enteric smooth muscles (Yamada *et al.* 2002) and hepatocyte-like cells (Ishizaka *et al.* 2002) *in vitro*. Further, we recently found that ES cells developed into hepatocyte-like cells after intrasplenic transplantation in mice that received short-term treatment with CCl_4_ (Moriya *et al.* 2007). In that study, it was also shown that intrasplenic transplantation of ES cells reduced fibrosis of the liver at the end of the 4-week period of CCl_4_ treatment.

In this study, we transplanted ES cells genetically labelled with enhanced green fluorescent protein (GFP) into the spleens of mice under long-term treatment with CCl_4_ to examine their potential efficacy toward liver fibrosis. Green fluorescent protein-immunopositive cells were consistently found in the areas of fibrosis in the mouse livers and liver fibrosis was reduced in mice that received ES cells, as compared with those that received phosphate-buffered saline (PBS). A second intrasplenic transplantation of ES cells at 12 weeks after the initial transplantation enhanced the improvement of liver fibrosis. Further, metalloproteinase 9 (MMP9)-immunopositive cells derived from ES cells were found in the livers of group 1 mice, as well as those derived from the recipients themselves, which were immunopositive for DlK-1, a hepatoblast marker (Tanimizu *et al.* 2003). These results suggest the potential usefulness of ES-based cell therapy for liver fibrosis and that improvement of CCl_4_-induced liver fibrosis by transplantation of ES cells may be due to the emergence of metalloproteinase-producing hepatoblast-like cells.

## Materials and methods

### Murine ES cell line

We utilized G4-2 cells from a mouse ES cell line (129/SvJ mouse ES cells, a kind gift from Dr Hitoshi Niwa, RIKEN Center for Developmental Biology, Kobe, Japan), which carry an enhanced GFP gene under the control of the CAG expression unit (Niwa *et al.* 2000). For this study, undifferentiated G4-2 cells were maintained on gelatin-coated dishes without feeder cells in Dulbecco’s modified Eagle’s medium (Sigma, St Louis, MO, USA) supplemented with 10% foetal bovine serum (FBS; GIBCO/BRL, Grand Island, NY, USA), 0.1 mM 2-mercaptoethanol (Sigma), 10 mM non-essential amino acids (GIBCO/BRL), 1 mM sodium pyruvate (Sigma) and 1400 U/ml of leukaemia inhibitory factor (GIBCO/BRL). The G4-2 cells carried the blasticidin S-resistant selection marker gene driven by the Oct-3/4 promoter (active under undifferentiated status) and were occasionally cultured in medium containing 10 μg/ml blasticidin S to eliminate differentiated cells.

### Preparation of graft cells and transplantation

Culture dishes (9 cm in diameter) were used to maintain the undifferentiated ES colonies, then washed with 8 ml of ice-cold PBS (pH 7.4) thrice and treated with 1.0 ml of 0.025% trypsin/PBS for 2 min at 37 °C. Five millilitres of ES maintenance medium containing 10% FBS was added to each dish to stop trypsin activity. Single cell solutions were easily obtained by repeated pipetting. Cells were washed with ice-cold PBS thrice and finally prepared for transplantation in a PBS solution at a cell concentration of 1 × 10^6^/ml.

Green fluorescent protein-positive undifferentiated ES cells at 1 × 10^5^ (0.1 ml of 1 × 10^6^/ml solution) were transplanted into the spleen, using an intrasplenic transplantation procedure previously described (Moriya *et al.* 2007). Briefly, a 1-cm incision was made on the left flank and the spleen was extracted through the incision. Then, 0.1 ml of the graft cell solution (1 × 10^6^ cells/ml) was slowly infused into the inferior pole of the spleen using a 31-gauge needle and a Hamilton syringe. Once the infusion was complete, the syringe was left in place for 1 min.

### CCl_4_ treatment

To induce liver damage, CCl_4_ at 0.5 ml/kg of body weight was injected into the peritoneum twice a week throughout the treatment period.

### Grouping of animals and outline of experiments

Female C57BL/6 mice were purchased from Japan SLC (Shizuoka, Japan) at 6 weeks of age and used as experimental animals. All procedures including the surgical steps were performed in accordance with the Guidelines of Nara Medical University for experiments involving animals and recombinant DNA.

In experiment 1, 56 mice received CCl_4_ treatment for 4 weeks, after which four were killed and fibrosis of the livers was examined, with that day was designated as day 0. The remaining 52 were divided into group 1 (*n*= 28) and group 2 (*n*= 24). Group 1 mice received transplantation of 1 × 10^5^ graft cells (0.1 ml of 1 × 10^6^/ml solution) on day 0, while group 2 mice were injected with 0.1 ml of PBS, instead of graft cells. Mice in both groups were treated with CCl_4_ twice a week consecutively. Liver sampling was performed after 1, 2, 4, 8 and 12 weeks (W1, W2, W4, W8 and W12 respectively) using four mice from each group, as well as on day 1 using group 1 mice only.

**Figure 1:**
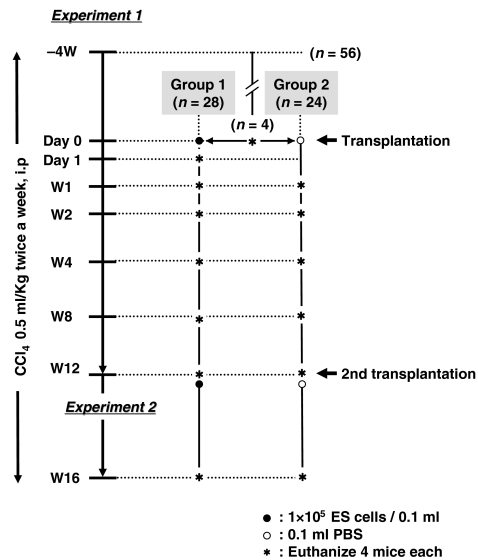
Grouping of experimental animals and outline of the experiments. Female C57BL/6 mice (*n*= 56) received CCl_4_ treatment for 4 weeks, after which four mice were killed, which was designated as day 0. The remaining 52 mice were divided into group 1 (*n*= 28) and group 2 (*n*= 24). Group 1 received transplantation of 1 × 10^5^ graft cells on day 0 and group 2 were injected with 0.1 ml of PBS. Liver sampling was performed at W1, W2, W4, W8 and W12 using four mice from each group, as well as on day 1 in group 1 only. At W12, the remaining four mice in group 1 received a second transplantation of graft cells and those in group 2 received 0.1 ml of PBS into the spleen. All were then subjected to an additional 4 weeks of treatment with CCl_4_.

In experiment 2, the remaining four mice in group 1 received a second transplantation of graft cells into the spleen at W12, while those in group 2 received 0.1 ml of PBS, then both groups continued to receive treatment with CCl_4_ for an additional 4 weeks. Animal grouping and an outline of the experiments are shown in Figure 1.

### Tissue preparation

The livers were thoroughly perfused via the heart with 4% paraformaldehyde to wash out contaminating blood cells. For fixation, the perfused livers were incubated with 4% paraformaldehyde overnight and then soaked in 30% sucrose for 3 days. Tissues were frozen on dry ice and sectioned into 5-mm slices using a cryostat in preparation for staining.

### Staining for collagen using picro-Sirius red

Frozen tissue sections were desiccated completely, then washed with distilled water for 5 min and incubated for 40 min in a saturated picric acid aqueous solution (1.1 g of picric acid in 100 ml distilled water). Next, a 0.1% concentration of picro-Sirius red powder was mixed into the solution and tissue sections were additionally soaked for a few minutes. When the sections were determined to be properly stained, they were removed, and rinsed twice in 0.01 N HCl and distilled water. Finally, the sections were briefly dehydrated with 70% ethanol for 30 s and 100% ethanol for 1 min, fixed with xylene and placed under coverslips.

### Staining for collagen using Mallory-azan

First, frozen tissue sections were desiccated, then incubated in an azocarmine G aqueous solution (0.1 g of azocarmine G powder and 1 ml of absolute acetic acid in 100 ml of distilled water) at 60 °C for 60 min and then at room temperature for 30 min. Next, after rinsing in distilled water, the tissue sections were soaked in a 5% molybdenum phosphate aqueous solution overnight, after which they were rinsed in distilled water, and soaked in an aniline blue and orange G-mixed aqueous solution (0.5 g of aniline blue, 2 g of orange G and 8 ml of absolute acetic acid in 100 ml of distilled water) at room temperature for 60 min. Finally, the sections were dehydrated, fixed and placed under coverslips.

### Immunohistochemistry of GFP

Frozen tissue sections were desiccated completely, then washed with PBS and soaked in PBS containing 5% bovine serum albumin (BSA) for 5 min, after which they were incubated overnight with anti-GFP (1:1000; Nacalai Tesque Inc, Kyoto, Japan). The sections were then washed with PBS and incubated with biotin-conjugated anti-rat IgG secondary antibodies for 1 h at 37 °C (1:200; Vector Laboratories Inc, Burlingame, CA, USA). Next, for the purpose of intrinsic peroxidase elimination, the sections were immersed in 3% hydrogen peroxide (H_2_O_2_) for 30 min. The sections were incubated with Vectastain ABC® reagent (Vector Laboratories Inc) for 2 h at 37 °C, then treated with 0.02 M Tris–HCl buffer (pH 7.5) containing 4% diaminobenzidine tetrahydrochloride (Vector Laboratories Inc) in the presence of 0.0004% H_2_O_2_ for 2 min at room temperature. Stained sections were rinsed with distilled water and immersed in ethanol and xylene for complete dehydration. Finally, the sections were enclosed with Entellan-new® (Merck, Tokyo, Japan).

### Immunofluorescence analyses

Goat anti-MMP9 (1:100; Santa Cruz Biotechnology Inc, Santa Cruz, CA, USA), rabbit anti-F4/80 (1:100; Santa Cruz Biotechnology Inc), and either rabbit or rat anti-GFP (1:500; Molecular Probe Inc, Eugene, OR, USA or Nacalai Tesque Inc, Kyoto, Japan respectively) were used as primary antibodies. For the secondary antibodies, Alexa Fluor® 546 goat anti-rabbit IgG, Alexa Fluor® 488 goat anti-rat IgG or donkey anti-goat IgG, Alexa Fluor 350 goat anti-rabbit IgG and Alexa Fluor® 546 goat anti-rat IgG were purchased from Molecular Prove Inc and used at a final concentration of 1:200. Frozen tissue sections were desiccated completely at room temperature using a dryer, washed with PBS thrice for 5 min each and soaked in PBS containing 5% BSA once for 5 min, then incubated overnight with the primary antibodies. The sections were then washed with PBS thrice for 5 min each and incubated with the Alexa Fluor® conjugated secondary antibodies for 1 h at 37 °C, then washed thrice for 5 min each with PBS and mounted on glass slides from an Anti-fade Kit (Molecular Prove Inc). Positive cells in the liver were observed using a Provis microscope (Olympus, Tokyo, Japan) equipped with a charge coupled device camera. A total of 10 different areas in each liver section were analysed independently.

### Quantitative analysis of liver fibrosis

The degree of liver fibrosis was quantified by measuring the relative areas of fibrosis with the aid of computer software, as described previously (Watanabe *et al.* 2000). Briefly, areas were selected at random and assessed using meta-morph software (Universal Imaging Corporation, Downingtown, PA, USA) at a magnification of ×40. The mean value of six randomly selected areas per sample was used to evaluate the degree of liver fibrosis, with liver fibrosis on day 0 determined to be the standard value of fibrosis index (F.I.) 1.0.

### Statistical analysis

Data are presented as the mean ± SD. Differences between groups were analysed by Student’s *t*-test, with *P*< 0.05 considered to be significant.

## Results

### Presence of ES-derived cells in CCl_4_-treated livers

**Figure 2:**
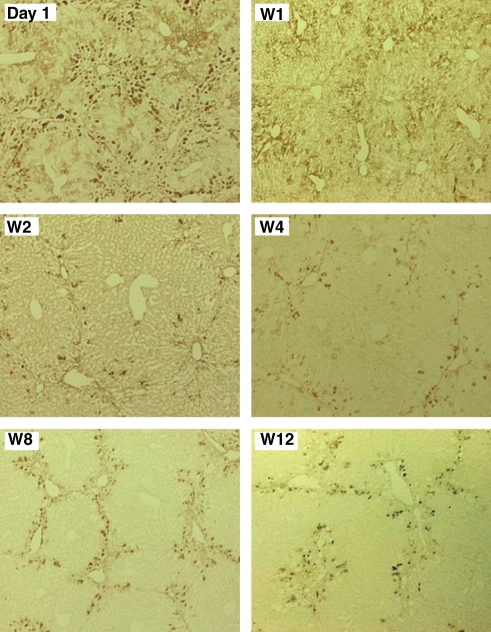
Presence of ES-derived cells in CCl_4_-treated livers. GFP-immunopositivity was examined in liver specimens from group 1 mice on day 1, then at W1, W2, W4, W8 and W12.

Transplantation was performed after pretreatment with CCl_4_ for 4 weeks. GFP-immunopositivity in the liver specimens was examined on day 1, as well as at W1, W2, W4, W8 and W12 in the group 1 mice (Figure 2). There were a number of GFP-immunopositive cells in the livers of group 1 mice on day 1. Thereafter, although the numbers of those cells decreased, they remained in the livers throughout the duration of the experiment. The distribution of immunoreactivity against GFP was observed predominantly in the periportal regions of the hepatic lobules.

### Decreased fibrosis following ES transplantation

**Figure 3:**
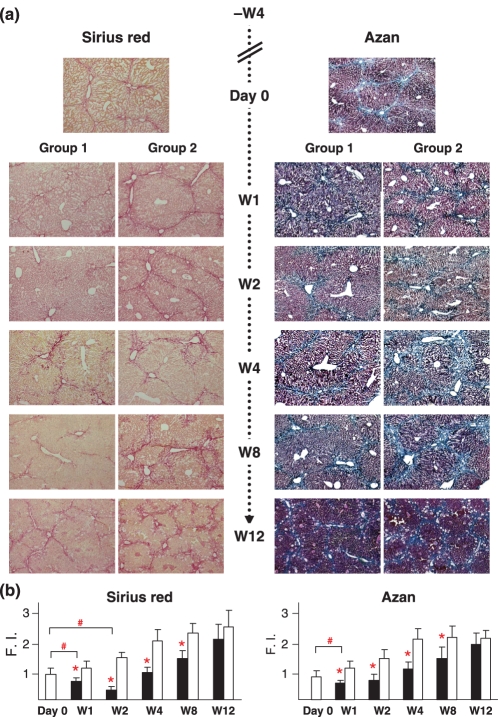
Decreased fibrosis by ES transplantation. (a) Liver histology findings on day 0, and at post-transplantation at W1, W2, W4, W8 and W12. Presumable liver fibrosis was induced by 4 weeks of treatment with CCl_4_. In group 2 mice, which received PBS, liver fibrosis continuously progressed, whereas that was reduced in group 1 mice, which received ES cell grafts. (b) Quantitative evaluation of liver fibrosis using image analyser. Fibrosis reduction was observed in group 1 (closed squares) as compared with group 2 (open squares) (*P< 0.05). In a comparison with liver fibrosis on day 0, reductions were confirmed at W1 and W2 in group 1 using picro-Sirius red staining and at W1 using Mallory-azan staining (^#^*P*< 0.05). Liver fibrosis on day 0 was determined to be the standard value of Fibrosis Index (F.I.) 1.0.

Liver fibrosis was evaluated histologically using picro-Sirius red and Mallory-azan staining. Pretreatment with CCl_4_ for 4 weeks caused considerable liver fibrosis (Figure 3a, top). In group 2 mice, which received PBS instead of ES cells, liver fibrosis continuously progressed following treatment with CCl_4_ (Figure 3a, group 2). When compared with group 2 mice, reduced liver fibrosis was observed in group 1 (Figure 3a, group 1). The results of quantitative evaluations using an image analyser are shown in Figure 3b. A significant reduction in liver fibrosis was observed in group 1 as compared with group 2 at all observation points except W12.

### GFP-immunopositive cells in locations neighbouring fibrosis areas

**Figure 4:**
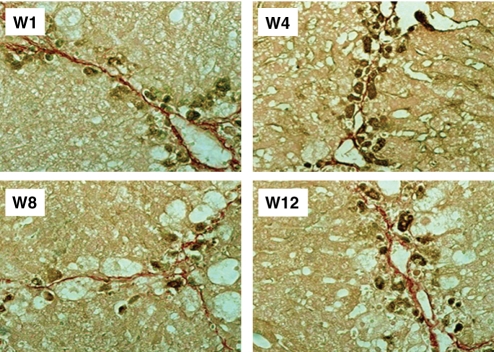
GFP-immunopositive cells in locations adjacent to fibrosis areas. GFP-immunopositive cells were found adjacent to or along with fibres in fibrotic areas, as shown by picro-Sirius red staining.

The location of GFP-immunopositive cells was determined using liver specimens from group 1 mice taken at W1, W4, W8 and W12 (Figure 4). GFP-immunopositive cells were found adjacent to or along with fibres, as shown by picro-Sirius red staining.

### Expression of MMP9 on hepatoblast-like cells in the livers

**Figure 5:**
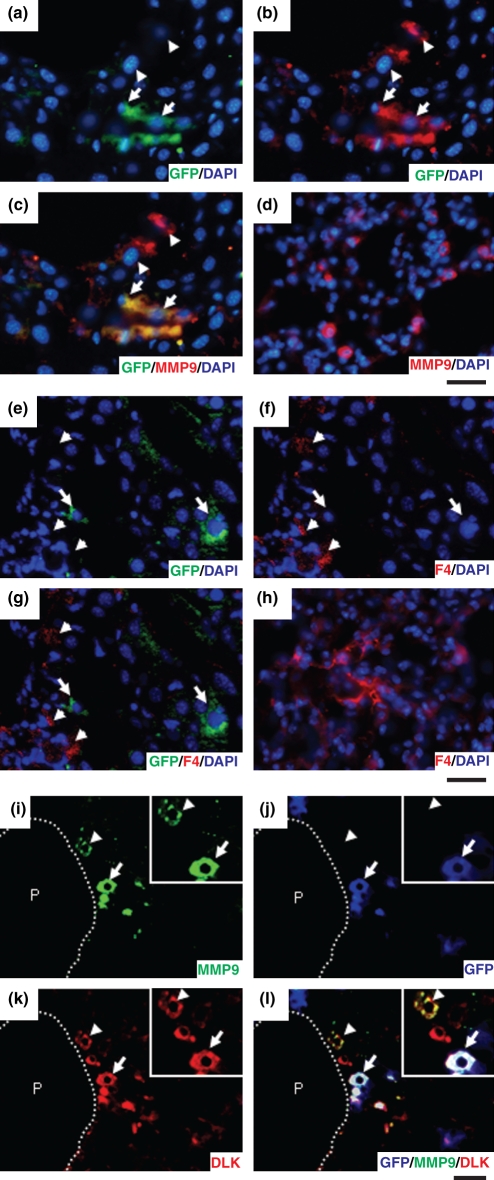
Immunofluorescence analyses. (a–d) GFP and MMP9-immunopositive findings were examined using liver specimens from group 1 mice taken at W12 (a–c) and specimens from normal C57BL/6 mice at 6 weeks of age (d). GFP-immunopositive cells were also MMP9-immunopositive (arrows), while some MMP9-immunopositive cells were not positive for GFP (arrowheads). Abundant immunopositivity for MMP9 was observed in the lung. Bar = 40 μm. (e–h) Immunopositivity for GFP and F4/80, a marker of macrophages, was examined using liver specimens from group 1 mice taken at W12 (e–g) and from normal C57BL/6 mice at 6 weeks of age (h). GFP-immunopositive cells were negative for F4/80 (arrows), while F4/80-immunopositive cells were not positive for GFP (arrowheads). Abundant immunopositivity for F4/80 was observed in the lung. Bar = 40 μm. (i–l) Immunopositivity for MMP, GFP and DlK-1 was examined using liver specimens from group 1 mice taken at W12. MMP-immunopositive cells (arrows and arrowheads, magnified in insets), derived from ES cells (arrows) and resident liver cells (arrowheads), were positive for DlK-1. P, portal vein; bar = 40 μm.

To explore the mechanism of fibrolysis, we examined the expression of MMP9 immunohistochemically using liver specimens from group 1 mice taken at W12 (Figure 5a–c). As expected, the transplanted cells expressed MMP9 (Figure 5a–c, arrows). Further, there were some cells immunopositive for MMP9 that were not immunopositive for GFP (Figure 5a–c, arrowheads), indicating that they had originated from the recipient mice. Our results suggest that not only transplanted ES-derived cells, but also endogenous fibrolysis activities contributed to improved liver fibrosis. Although MMP9 is known to be expressed in alveolar macrophages in the lung (Figure 5d,h), no expression of F4/80, a marker of macrophages, was found in GFP-immunopositive cells in the liver (Figure 5e–g arrows). There were some cells immunopositive for F4/80 in the liver, although they were not immunopositive for GFP (Figure 5e–g arrowheads).

We also investigated the expression of DLK to elucidate the characteristics of the cells that were MMP9-immunopositive and F4/80-negative. DLK is a molecule identified as a possible hepatoblast marker and known to be strongly expressed on hepatoblasts in mouse embryonic livers until embryonic day 16.5, after which it becomes downregulated and disappears by birth (Tanimizu *et al.* 2003). Interestingly, MMP9-immunopositive cells, irrespective of their origin from graft cells or the recipient, were immunopositive for DlK-1 (Figure 5i–l arrows or arrowheads respectively), indicating that they were hepatoblast-like cells.

### Liver fibrosis improved by secondary ES cell transplantation

**Figure 6:**
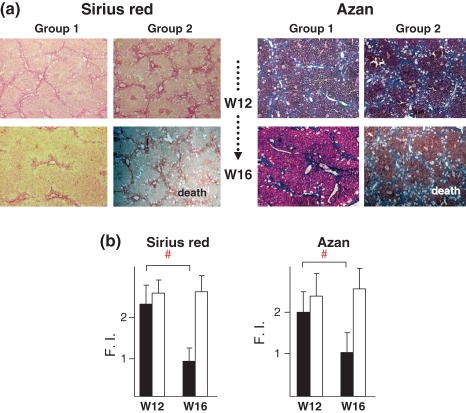
Liver fibrosis improved by second ES cell transplantation. (a) Liver histology results for group 1 mice at W16 and group 2 at death. At W12, the remaining four mice in each group received a second transplantation of ES cells (group 1) or PBS (group 2) into the spleen. The mice in group 1 survived until W16, whereas all in group 2 died by W15. (b) Quantitative evaluation of liver fibrosis using image analyser. Liver fibrosis was improved at W16 in group 1. Closed and open squares show group 1 and group 2 respectively. Liver fibrosis on day 0 was determined to be the standard value of Fibrosis Index (F.I.) 1.0.

At W12, the remaining four mice in group 1 received a second transplantation of ES cells into the spleen, while those in group 2 received 0.1 ml of PBS, after which mice in both groups were subjected to an additional 4 weeks of treatment with CCl_4_. However, all mice in group 2 died no later than W15 and exhibited ascites before death, leading us to conclude that the cause of death was liver failure. The livers were isolated within 8 h after death and histology findings for group 1 mice at 4 weeks after the second transplantation (W16) showed reduced fibrosis as compared with W12 (Figure 6a), which was determined quantitatively by image analysis (Figure 6b). In group 2, histological findings of the liver showed advanced fibrosis with lipid accumulation, although there was no significant difference between W12 and W16.

### Tumour formation

All of the livers in the experimental animals were removed and the lobes sliced into 2-mm widths, with specimens from the middle lobes used for microscopic analysis. No tumour formation was observed macroscopically or microscopically in any of the livers and spleens. Organs other than the liver and lungs were found to be macroscopically intact.

## Discussion

Recently, transplantation of bone marrow cells (Sakaida *et al.* 2004), bone marrow stem cells (Wu *et al.* 2005), bone marrow mesenchymal stem cells (Fang *et al.* 2004; Zhao *et al.* 2005; Oyagi *et al.* 2006) and foetal liver progenitor cells (Zheng *et al.* 2006) has been reported to reduce liver fibrosis. However, the effect of ES cells on liver fibrosis has not been reported. We previously reported that ES cells transplanted into the spleens of CCl_4_-treated mice migrated to the liver and differentiated into hepatocyte-like cells (Moriya *et al.* 2007). In that study, we also noted the possibility that ES cells might reduce fibrosis of the liver, although the total experimental period was only 5 weeks, which consisted of a 1-week pretreatment with CCl_4_ before intrasplenic transplantation of ES cells and then an additional 4 weeks of treatment. In this study, we conducted a long-term experiment to examine the effects of ES cell transplantation on liver fibrosis. Mice were pretreated with CCl_4_ for 4 weeks to produce considerable liver fibrosis prior to transplantation, then fibrosis was observed histochemically for up to 16 weeks after the initial transplantation.

As expected, obvious fibrosis was induced in the livers by pretreatment with CCl_4_. First, we determined whether the transplanted ES cells migrated to and were retained in the liver for a sustained period, because we noted only few grafted cells at 30 days after transplantation in mouse livers in a previous experiment (Moriya *et al.* 2007). In this study, we found a considerable number of transplanted cells in the liver even at W12. These contrasting results were probably because of the length of the CCl_4_ pretreatment protocol, which consisted of two intraperitoneal injections over 1 week in the previous study, whereas we gave eight injections of CCl_4_ over a 4-week period in this study. We considered that a more favourable microenvironment might be induced in the liver by the present repeated CCl_4_ administration, under which ES cells were able to migrate to and reside in the liver. Recently, the CXCR4/SDF1 system has been suggested to be involved in the migration of bone marrow cells to inflamed organs (Dalakas *et al.* 2005), including CCl_4_-treated livers (Kollet *et al.* 2003; Jung *et al.* 2006; Son *et al.* 2006). Although the involvement of the CXCR4/SDF1 axis in hepatic migration of ES cells transplanted into the spleen is unknown, we noted a much higher level of SDF1 expression immunohistochemically in the livers of mice pretreated with CCl_4_ for 4 weeks as compared with those pretreated for 1 week (data not shown).

These results demonstrated that transplantation of ES cells improves liver fibrosis, while immunohistochemistry findings also revealed GFP-immunopositive cells in locations neighbouring areas of fibrosis at all observation time-points. Bone marrow cells and bone marrow-derived mesenchymal stem cells have been reported to migrate to fibrosis areas of the liver and show activities of matrix metalloproteinases after transplantation (Kollet *et al.* 2003; Sakaida *et al.* 2004; Higashiyama *et al.* 2007). It is conceivable that ES cells elicit fibrolysis activity similar to that of bone marrow cells. As expected, ES-derived cells in the present livers were immunopositive for MMP9, some of which were negative for GFP. These findings suggest that the improvement in liver fibrosis after transplantation of ES cells was a result of fibrolysis activities by both ES-derived and recipient-derived cells. Next, we attempted to identify the nature of the MMP9-immunopositive cells. As the GFP-immunopositive cells were negative for F4/80, we considered that the ES-derived MMP9-immunopositive cells were also negative for F4/80. Interestingly, MMP9-immunopositive cells, irrespective of their origin from ES cells or the recipient, were immunopositive for DlK-1, a hepatoblast marker (Tanimizu *et al.* 2003), suggesting that hepatoblast-like cells play an important role in the regression of liver fibrosis. Investigation of the pathophysiology of the generation of MMP9-expressing hepatoblast-like cells from ES cells and liver resident cells in CCl_4_-injured livers may lead to discovery of a new treatment approach for liver fibrosis.

We performed a second transplantation of ES cells at W12, which led to an additive effect for improved fibrosis seen at W16. All four mice in group 1 that received ES cell transplantation twice, day 0 and W12, survived to the final observation point at W16. However, none of the mice that received PBS instead of ES cells at the same time-points survived to W16, but rather showed accumulated ascites, leading us to conclude that the cause of death was liver failure. Thus, these findings suggest the potential usefulness of ES cell transplantation to treat liver cirrhosis.

Recently, a novel trial of intravenous transplantion of autologous bone marrow-derived stromal cells (BMSCs) in patients with liver cirrhosis reported improved serum albumin and total protein levels (Terai *et al.* 2006). In contrast to that method, the present ES cells used as grafts and host mice were an allogeneic combination, as the cells were derived from 129SvJ mice and transplanted into C57BL/6 mice. In the future, it is considered that human ES cells used in therapeutic strategies will likely be allogeneic to the host, except in cases where ES cells are produced with a nuclear transfer technique using nuclei from the host cells (Lavoir *et al.* 2005 or by introducing four recently identified genes into host somatic cells (Takahashi *et al.* 2007; Yu *et al.* 2007). Although a high incidence of teratomas in livers was reported following syngeneic transplantation of undifferentiated ES cells and differentiating EBs into C57BL/6 mice receiving CCl_4_ treatment (Chinzei *et al.* 2002; Yamamoto *et al.* 2003), we found no tumours in the livers of mice that received undifferentiated allogeneic ES cells during the 16-week experimental period.

In conclusion, our results showed that undifferentiated ES cells grafted into the spleen migrated to the liver and reduced liver fibrosis in mice chronically treated with CCl_4_. For the coming era of controlled cell growth, ES cells are a promising cell source for treatment of liver cirrhosis.
